# Balancing diversity and connectivity in multi-level governance settings for urban transformative capacity

**DOI:** 10.1007/s13280-018-01142-1

**Published:** 2019-02-15

**Authors:** Sara Borgström

**Affiliations:** Department of Sustainable Development, Environmental Science and Engineering, The Royal Institute of Technology, 100 44, Stockholm, Sweden

**Keywords:** Collaboration, Decentralisation, Governance context, Local sustainability initiatives, Trans-disciplinary research

## Abstract

Transformation towards sustainable development is about findings new ways of thinking, organising and doing to navigate wicked challenges such as climate change and urbanisation. Such challenges call for new governance modes that match the complexity of the systems to be handled, where multi-level governance and collaborative approaches have been suggested to contribute to such transformative capacity building. This in-depth, trans-disciplinary study investigates how the multi-level governance context in Stockholm, Sweden, influences the transformative capacity from the perspective of local sustainability initiatives. It was found that even though the decentralized governance of the Stockholm region hosts a great potential in supporting city wide transformation, it is hampered by disconnect between actors, levels and sectors and the short-term funding structure. The suggested interventions highlight the tension between enabling collaborations, while safeguarding a high local diversity of initiatives and flexibility to ensure sustained space for innovation and learning.

**Electronic supplementary material:**

The online version of this article (10.1007/s13280-018-01142-1) contains supplementary material, which is available to authorized users.

## Introduction

Today’s world is understood as consisting of nested complex systems and needing complex governance to match. This becomes evident in any attempt for sustainable development, which is inevitably about handling formidable challenges where social, ecological and economic complexities are intertwined across scales (e.g., climate change and urbanization). The incapability of many present-day decision-making structures and processes (i.e., governance systems) to match global complexity is an important reason for unsustainable pathways (Meadowcroft 2002; Cumming et al. 2006; Bodin 2017; Webb et al. 2018). Governance is here defined as the institutions and processes that direct interactions between diverse actors representing many different interests, forms of power and scales (Healey 2008).

The complexity of governance has been increasingly acknowledged, inspiring the development of multi-level governance (MLG) conceptual model for describing and analyzing this complexity in terms of both structures and processes (Hooghe and Marks 2003; Stephenson 2013). The MLG model acknowledges that multiple actors interact, and both formal power and informal power are dispersed across levels and sectors (Geels 2002; Kooiman 2003). These interactions impact the direction, as well as the execution, of decisions, such as what knowledge is used, whose interests are recognized and what actions are prioritized (Kooiman 2003). This paper deals primarily with the state and society interaction as one of the three dimensions of MLG presented by Piattoni (2009). Furthermore, it engages with the coordination dilemma between an increasing number of overlapping jurisdictions in sustainability governance and especially the effects of so called second order coordination costs (Hooghe and Marks 2003). These costs emerge from need of coordination among institutions aiming at coordinating human activities (ibid).

Within sustainable development policy and practice, cross-scale interactions have been captured in various attempts to engage with local actors and, therefore, recognize the links between local places and practices and the dynamics of larger geographical scales. For example, balancing top-down decision making with bottom-up perspectives, along with different approaches for enhancing participation, collaboration and inclusion (e.g., Armitage et al. 2009; Newig and Fritsch 2009; Pahl-Wostl 2009; Satterthwaite 2013) have recently developed into trans-disciplinary knowledge co-creation processes (Lang et al. 2012; Voorberg et al. 2014; Frantzeskaki and Kabisch 2015). Though learning from local actors’ ways of thinking about, organizing and doing sustainable development is important and holds great potential, to contribute to larger-scale transformation toward sustainable development it is still necessary to better understand the contextual factors that have an impact on this potential (Avelino and Wittmayer 2016; Frantzeskaki et al. 2016). To be able to support situated agency of sustainable development, it is instrumental to understand the arena where it plays out.

Cities are complex systems in which the urban MLG is a key dynamic for addressing local to global sustainable development challenges (McCormick et al. 2013; Wolfram et al. 2016; Ehnert et al. 2018a). Cities are also learning laboratories with an institutional thickness, and therefore, they are potential leaders in transformative processes toward sustainable development (Nevens et al. 2013; Bulkeley et al. 2016; Voytenko et al. 2016; Wolfram 2016a). Local urban initiatives have been found harboring an, often underused, knowledge about and ability to foster alternative ways to activate city-scale transformation (Seyfang and Smith 2007; Frantzeskaki and de Haan 2009). Therefore, an important piece of urban sustainable development is to increase understanding about how the urban MLG structures and processes hinder or enable the emancipation of this local, urban potential.

The characteristics of urban local initiatives in relation to sustainable development and transformation have been investigated in several, often sector specific, case studies—for example, considering energy (Seyfang and Haxeltine 2012) or green space (Svendsen and Campbell 2008) or focusing on the initiatives’ internal dynamics instead of contextual or relational factors (Hodson and Marvin 2012). McCormick et al. (2013) emphasize that sustainable urban transformation is mostly a social endeavor where new ways of governing, planning and engaging a diversity of key stakeholders are central. Similarly, studies about civil society’s local initiatives (grassroots-level actors) point to the need for new modes of governance (Seyfang and Haxeltine 2012; McCormick et al. 2013; Frantzeskaki et al. 2016; Gorissen et al. 2017; Wolfram 2016a; Webb et al. 2018). They highlight not only empowerment, but also the need for higher-level coordination that balances initiative autonomy with improved inter-initiative connectivity (Buijs et al. 2016; Baibarac and Petrescu 2017; Gorissen et al. 2017) and intermediaries acting as bridges between levels and sectors (Folke et al. 2005; Hamann and April 2013; Wolfram 2016a).

At the conceptual level, this is a balancing act between supporting diverse ways of thinking, organizing and doing while promoting purposeful connectivity (e.g., exchange of information, resources and support), both of which have been identified as important contributors to resilience (Biggs et al. 2012) and transformative capacity (Wolfram 2016b). Diversity of options and the ability to continuously experiment and, therefore, learn and have the capacity to adapt to new circumstances, are at the core of resilience and the capacity to handle change (Biggs et al. 2012). This is something transformation toward sustainable development must include (Wolfram 2016b). However, diversity might also cause restrictions of local, small-scale projects and pilots due to limited or lack of connectivity, which for instance risks to hamper pathways for upscaling (Smith and Raven 2012). In particular, the MLG setting requires multi-facetted connectivity for coordination across levels, ensuring awareness of how larger and smaller scales interact, and to ensure inclusion and broad participation. The most common approaches involve promoting and supporting various forms of broad participation, cooperation, collaboration, co-management, co-creation, etc. (e.g., Bodin 2017). However, connectivity might also threaten diversity since intense exchange over a long time can lead to increased homogenization and can also direct limited resources toward communicating and negotiating instead of acting.

The overarching aim for this study is to investigate how an urban MLG context influences the transformative capacity of a city from the perspective of local sustainability initiatives (LSIs) that can lead by private, public or civic actors. The rational is the importance of understanding how different actors navigate the MLG context and how that affects their capacity to act for change locally and for the overall system transformative capacity. More specifically, the study focuses on the enabling and inhibiting factors for the existence, sustenance and potential city- scale impact of LSIs. Based on the trans-disciplinary process of developing a road-map for transitioning to sustainable development in the urban, MLG context of Stockholm region, Sweden, the paper suggests how the existing potential of these LSIs can be better acknowledged, captured and developed in Stockholm’s city-wide sustainability work. The proposed strategies are then discussed from an MLG perspective with special focus on the inherent tensions between diversity and connectivity.

## Materials and Methods

The paper’s empirical basis is a qualitative, trans-disciplinary case study in which a mixed set of methods—including interviews, focus group discussions and workshops—were triangulated, and preliminary results were iterated with different actors (sensu Creswell 2015). The trans-disciplinary process concluded with co-production of a roadmap with suggestions for moving toward sustainable development in the Stockholm (Borgström and Oreskovic 2016).

### Stockholm MLG context

The case study is part of a larger research project called Accelerating and Rescaling Transitions to Sustainability (ARTS), which ran from 2014 to 2016 and included five city-regions in Europe (European Commission 2017). The Swedish national system is relatively decentralized when compared with, for example, the UK unitary and centralized, and German federal, governance systems (Ehnert et al. 2018a) and where the local level, the municipalities, have strong formal power and responsibility for many public services and interests. This localization of power has a long history in Sweden and started before the more recent decentralization reforms in Europe and elsewhere (Hooghe and Marks 2003). The idea behind self-governing municipalities is that they enable local democracy by placing short ‘distances’ between residents and decision-makers, as well as delegating responsibility to the local level where most issues are experienced (Nilsson and Forsell 2013). In addition and related to this decentralization, Sweden has a long tradition of involving civic associations in formal decision-making processes which reaches more than a century back in time (von Essen et al. 2015). More recently, and potentially in response to increasing awareness of sustainability challenges, local engagement has taken new forms; new initiative networks have been established, and the public sector emphasizes the need for new ways to address sustainable development.

The study’s geographical scope is Stockholm County, which, with 2.3 million inhabitants, is Sweden’s most urbanized region (SCC 2018). The urbanization pressure is very high, and the population is estimated to increase to 3.4 million inhabitants by 2050 (ibid). Stockholm has the reputation of being a city well on its way toward sustainable development. For example, it was appointed as the first European Green Capital in 2010—a title which recognized the city’s achievements and its innovative approaches to environmental challenges (Metzger and Rader Olsson 2013). However, mimicking Sweden’s national trend, the Stockholm region does not fulfil any of the 16 National Environmental Quality Objectives (www.miljomal.se, retrieved 180920). The Stockholm region includes 26 municipalities, which is a large number compared to other larger Swedish cities such as Gothenburg and Malmö. The municipalities in Stockholm differ in size, demographics, economies, political priorities and urbanization strategies. They are led by locally elected politicians, and the economies are based on local tax income, which, in turn, depends on the municipal population size and the residents’ income levels. On the other hand, being part of a metropolitan region means that the municipalities share certain traits, such as physical landscape, urbanization pressure, technical infrastructure, labor markets, larger scale environmental and sustainability challenges, as well as the national governance context. The regional authorities have a guiding role—primarily the Stockholm County Administrative Board, which is responsible for implementing national regulations and policies, and the Stockholm County Council, which regularly produces regional development plans and is responsible for healthcare and public transportation (SCC 2018). The need for coordination between the municipalities is growing, and not the least when sustainability challenges are to be addressed, such as food and water security, ecosystem services provision and climate change mitigation and adaptation (Nykvist et al. 2017; SCC 2018). The multi-levelness and high degree of decentralization make the Stockholm region an interesting and relevant case for disentangling the roles of local sustainability initiatives (LSIs) in the transformative capacity of urban, MLG settings.

### Identifying and investigating local sustainability initiatives

For this research project, local sustainability initiatives (LSIs) were defined as initiatives that are locally based, with activities aiming at transforming the society toward environmental sustainability. The LSIs engage in at least one, or a mix of several, of the following environmental sustainability dimensions: energy, transport and mobility, food, water, resource management, the built environment, nature conservation and restoration and education and knowledge development for sustainability. They are led by public, private or civic actors, or by a mix of these. This broad scope is motivated by the urge to move beyond a focus on certain local initiatives, such as the grassroots type, and to have a multi-domain approach, combining different sectors to better capture the links between them (Wolfram et al. 2016).

An initial scoping was conducted in which a gross list of Stockholm-based LSIs was created, based on the research team’s prior knowledge and on informal discussions with eight key informants, including Stockholm’s sustainability arena representing consultants, civil servants at regional authorities, and representatives from adult education and local initiative networks (Table S1, Appendix S1). This first scoping was complemented by a desk-based exploration resulting in a list of 53 LSIs that were then described according to their leadership, time of initiation, main sustainability domain, activities and geographical scale of activity. To enable an in-depth study about how local actors, interact with their governance contexts, a sub-selection of LSIs was also made. The criteria for this list required the selected LSIs to be presently active and to have been active for at least a year—involving more than one person or household, being innovative in their regional context (in this case the Stockholm region), not being solely policy-oriented (e.g., not policy implementation programs or ‘think tanks’) or communication platforms (e.g., not Web sites, blogs).

From the initial list, 31 LSIs fulfilled these criteria, and this shortlist was discussed with the international project team and compared to similar lists from the other study areas to identify gaps in coverage and search biases. To fill these gaps and ensure that no relevant LSIs were missed, the shortlist was also discussed with the key informants. Finally, to enable an in-depth study of LSI dynamics within the Stockholm governance context, another subset of 16 LSIs was selected (Table 1), which maximized the coverage of different sustainability domains as well as leadership types. Semi-structured interviews were conducted in 2015 with one to two representatives from all the initiatives—except one, which declined participation because of respondent fatigue from participating in other research projects. Most of the respondents were initiators and/or present leaders of their LSIs. The interviews lasted 45–120 min. They were recorded and documented in writing (see Interview Guide in Appendix S2). Insights regarding regional-level environmental governance in relation to the LSIs were captured in separate interviews with four experts selected because of their professional positions and experience being active in the Stockholm sustainability arena in various roles (Table S2, Appendix S1).

**Table 1:** List of local sustainability initiatives (LSIs) in the Stockholm region included in the interview study. For a more detailed description of each LSI see Appendix S1

ID	LSI name in Swedish/English translation	Sustainability domain	Lead sector	Initiated (ended)	Main activity	Respondents (number)	Type
AM	Mobilsamåkning Adelsö/Adelsö Mobile Carpooling	Transport/mobility	Civil society/Private	2012 (2015)	Providing a local carpooling service linked to overall local sustainability ambitions on the Adelsö island	The two leaders of the LSI, volunteers with deep engagement in the local community (2)	Single
StS	Stadsodling Stockholm/Urban Agriculture Network Stockholm	Food (gardening)	Civil society	2013	Establishing links between local gardening initiatives across Stockholm region.	The initiator and present coordinator of the network, on a voluntary basis. (1)	Networking
ES	Ekologisk mat Södertälje/Organic Food in Södertälje	Food	Public sector	2010	Increasing the proportion of organic and local food in public meals (schools, aged, hospitals) in Södertälje municipality.	The municipal civil servant with responsibility for the LSI as part of being the municipal lead dietary manager. (1)	Municipal
BM	Bondens marknad/Farmers Market	Food	Association of local farmers	2000	Shortening the distance between local food producers and urban customers by arranging farmers markets.	One of the initiators of the LSI in Uppsala and Stockholm, on a voluntary basis. (1)	Regional
EA	Ekoodling Akalla/Hjulsta/Organic Gardening Akalla/Hjulsta	Food (gardening)	Civil society	2010	Urban gardening for local food provisioning in the peri-urban context of Järvafältet (NW Stockholm municipality), while including marginalized people (unemployed, immigrants, aged people).	Two of the initiators and present leaders of the LSI, on a voluntary basis.(2)	Single
HH	Hållbara Hökarängen/ Sustainable Hökarängen	Multiple	Private company/Public sector	2012 (2015)	City district (Hökarängen) wide initiative to support local sustainability lead by the rental housing company owned by Stockholm municipality.	Sustainability strategist at the Stockholmshem company and project leader of HH.(1)	Municipal
HP	HOPP! (Hökarängen Omställning och Permakulturprojekt)/Hökarängen transition and perma culture project	Multiple	Civil society	2014 (2016)	Transition movement in Hökarängen city district in Stockholm municipality aiming at transformation to a more sustainable lifestyle focusing on food security.	One of the three initiators and leaders of the LSI, on a voluntary basis.(1)	Single
OV	Omställning Värmdö/Transition Movement Värmdö	Multiple	Civil society	2011	Transition movement in Värmdö municipality aiming at transform into a more sustainable lifestyle.	One of the two initiators and leaders of the LSO, on a voluntary basis.(1)	Single
RF	Refo/Refo – Remake and Reuse of Clothes	Resource management	Private company	2012	Collecting textile waste for remake and reuse while supporting poor families and unemployed.	The initiator, leader and owner of the company. (1)	Regional
LT	Lekträdgården – Lek Odla Väx at Rosendal Trädgård/The Play Garden – Play Grow Cultivate at Rosendal Trädgård	Education/food	Foundation	2012	Providing gardening classes for kids from the Stockholm municipality.	The CEO of the foundation and one of the LSI leaders. (2)	Single
SN	Storstockholms naturguider/Greater Stockholm Nature Guides	Education/nature conservation	Civil society	2003	Providing nature guided tours in the green structures of Stockholm region for the public.	The present leader of the LSI. (1)	Regional
MV	Miljöverkstan Flaten/The Environmental Workshop Flaten	Education/nature conservation	Civil society	2013	Supporting young people to engage in their local landscape for long-term sustainability.	One of the initiators and present leader of the LSI. (1)	Single
KR	Rösjökilen samverkan/Rösjö Green Wedge Collaboration	Nature conservation	Public sector	2006	First initiative for collaboration around one of the green wedges in Stockholm region (NE parts)	One of the municipal civil servants involved in the initiation and establishment of the collaboration. (1)	Inter-municipal
KS	Mellankommunal samverkan kring gröna kilarna/Municipal Collaboration of Stockholm Green Wedges	Nature conservation	Civil society, Public sector	2006	Supporting establishment of multi-actor collaborations around the regional green wedges in the Stockholm region.	One of the initiators and present coordinator of the collaborations. (1)	Regional
GV	Ny grön våg/New Green Wave	Nature conservation	Civil society	2012	Establishing links between local initiatives defending local green structures against densification.	One of the coordinators of the network. (1)	Networking
X	HS2020^a^	Multiple	Civil society	2011	Citizen led engagement to fulfil the environmental ambition in the Hammarby Sjöstad city district in Stockholm municipality.	na	na

^a^Declined to participate due to involvement in a very recent and similar research project

All interview written documentation was reread while highlighting sections relevant to the aim and research questions. For an overview of the collected data, the interview content was shortened into coherent sentences and statements that gave relevant information—so-called meaning-bearing units (MUs) (Kvale 1996). MU analyses were iterative and steered by analytical components derived from the following analytical questions:

- What are the success factors that enable the LSIs?
- What structures and processes hinder or limit the LSIs?
- What are the identified needs for better using the LSI capacity?

During the first round of analysis, a preliminary set of codes were inductively complemented with new/refined codes based on the material (Appendix S3). The main themes captured descriptions of the LSIs (e.g., origins, aims, activities, present needs, geographical range of activities/members), factors affecting LSI progress and impact in the region (e.g., resources, flexibility, competition, drainage of capacity, political support). Beyond the scope of this paper, also other aspects of the LSIs interaction with the governance context were captured, the so-called transition acceleration mechanisms (Frantzeskaki et al. 2015; Ehnert et al. 2018b).

### Trans-disciplinary process toward a roadmap

The trans-disciplinary research design (Lang et al. 2012) aimed at contributing to the transformative capacity by involving and interacting with a large diversity of actors—representing different roles, administrative levels and sustainability domains—and, therefore, combining experience-based, practical, policy-oriented and research-based knowledge spheres (Tengö et al. 2014; Polk 2015; Webb et al. 2018). The process started with formulating key themes of importance to the Stockholm region’s transformative capacity. These themes, concerning enabling and hindering factors of LSIs in the Stockholm MLG setting, were based on the interview data and were initially formulated by the research team. They were then iteratively developed during three focus group meetings and two workshops in which different sets of actors participated (Appendix S1).

At all meetings, the participants were asked what other actors they thought should be invited into the process, and, hence, the list of potential participants for meetings and workshops grew over time. This was also a way, not only to broaden perspectives beyond the 15 LSIs and five experts included in the interview study and anchor the roadmap process, but also to secure meeting participation, since not all listed actors were able to attend all meetings to which they were invited (Appendix S1). In addition, and as part of the ARTS project setup, five independent artists with documented interest in sustainable development were included in a parallel dialogue process and participated in the two workshops. Professional collaborative process designers and facilitators (communicators and artists) were consulted to support the research team in the co-production process—navigating diversity among participants (i.e., differing knowledge, agendas, interests) and building trust and engagement in the process (Wittmayer and Schäpke 2014). After each meeting/workshop, a written summary was sent out to all invited participants, as well as information about the next steps.

In the first workshop (WS1, Appendix S1) all but four of the LSIs involved in the in-depth interview study participated (Table 1), but none of the experts were able to participate. The participants were asked to reflect upon and discuss the preliminary findings from the interviews presented by the research team. A diverse set of practical dialogue tools was used to support exchange of experiences and networking between the participants and to provide different ways of communicating insights to the research team and the other participants, e.g., own reflections that were noted down and then shared in pairs of participants, rounds of short presentations of insights in small groups for listening and reflection, and a collaborative task to present a joint narrative of the LSI situation in Stockholm as an artistic figuration. The research team documented the discussions in writing to complement the participants’ own notes and drawings.

Reflections from the public sector were captured during two focus group meetings (FG1 and FG2, Appendix S1) with representatives from municipalities and regional authorities, respectively. The participants were asked to reflect upon the presented preliminary findings based on their respective roles within the governance context. Then, ideas for the roadmap and possible ways to better use the LSIs’ local capacities were discussed. In preparation for the second workshop (WS2), a focus group with LSIs (both involved in the interview study and other LSIs invited by them, FG3, Appendix S1) was arranged to collect insights and ideas for the content and format of the roadmap co-production (e.g., what target group, what focus and what format). The focus group meetings were facilitated by two researchers who presented the findings and documented the discussions in writing.

The second and final workshop began with the identified hindering/enabling factors and potentials in the Stockholm region’s MLG, and, from that background, it developed and discussed strategies to be included in the roadmap for transformation toward sustainable development. All participants in previous steps of the project were invited to this workshop, as well as other key actors in the Stockholm region’s sustainable development arena (Appendix S1). The dialogue methods used included mini-interviews, graphical documentation, body interpretations and narrative exercises in groups. The research team again took documented the discussions in writing to complement the participants’ own notes and drawings. In the final steps, the roadmap was formulated as a written report and was circulated to all participants for online feedback.

## Results

### A diversity of LSIs

**Fig. 1:**
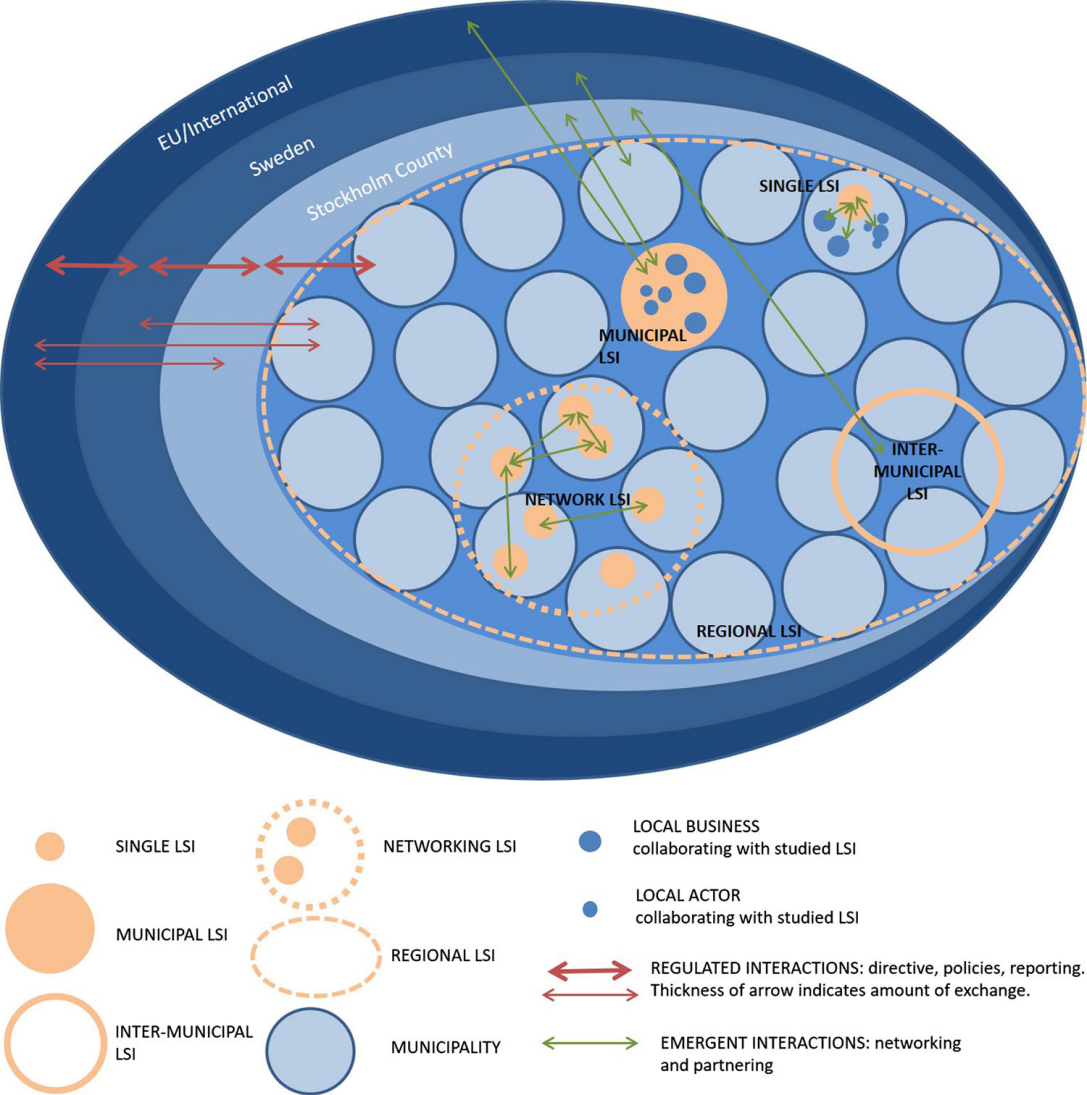
The multi-level governance structure and interactions in the Stockholm region. By M.S. Almqvist

Five LSI types were identified by the different ways they relate to the governance structure. The ‘single LSI’ works locally, within one municipality or municipal district, and is often connected to a local network of actors; the ‘municipal LSI’ is led or coordinated by a municipality; the ‘inter-municipal LSI’ aims to facilitate collaboration across municipal borders; the ‘networking LSI’ links together several small-scale initiatives; and the ‘regional LSI’ has activities across the whole region (Fig. 1, Table 1). This diversity of LSIs can be related to the decentralized and thereby diversified MLG structure of the Stockholm region, where each LSI has been initiated based on the municipal context(s), is dependent on, must adapt to and navigate this specific municipal context(s). This diversity is an important source of alternative solutions to local as well as regional sustainability challenges in the region. However, it also implies that there is no blueprint in how to best interact and engage with these initiatives and make use of their expertise. Furthermore, it means that structures and processes set up to support these initiatives must be flexible enough to include the large variation of initiatives. Although all LSIs are unique in their origin, evolution and approach, several commonalities were agreed upon during the trans-disciplinary discussions, and those are the focus of the following presentation of hindering and enabling factors.

### Municipal approaches

The municipalities have an important role in recognizing and supporting LSIs and also in acknowledging them toward the regional level. However, due to the high degree of decentralization, each of the 26 municipalities has its own approach to LSIs, and sustainable development generally. This was clear from the LSI interviews. For some LSIs, the municipal support was lacking, e.g., AM: *‘We have been working [on a] voluntary basis, and this is time consuming. There has been too much work, which has left no capacity for further development [of the initiative]. It would have been great with some municipal support. We contacted the municipality, but they did not really understand our initiative’.* For others, e.g., StS, it was partly a matter of lacking a contact point within the municipality: ‘*Everyone likes urban gardening in theory, but in practice it is confusing and there is no one at the municipality that you can communicate with regarding gardening*’. However, within the same municipality, but concerning other activities and in a specific district, MV was on the other hand met with enthusiasm: ‘*We contacted the City District Administration that was willing to [activate] change*—*“we have waited for you” they said. They are open to local initiatives not by top*-*down approach, but by supporting them as contributors to the community building in the district. They are smart in that they want to collaborate with the citizens’.*

Even when there is collaboration between an LSI and a municipality, the municipality might be willing to support piloting and projects, but not engage with the intention to change in more large-scale and long-term ways. OV experienced this, saying:

*‘When it never landed within the politics in three years’ time, then it is not productive, and you have to do something else. Those who have the money have not changed their perspective much. We did not realise how difficult it was to get them on-board. We could have started out from the understanding of a low interest within the municipality and assist them in taking small steps instead of running in there with too high ambitions of radical transition’.*

On the other hand, an important factor that contributes to the potential for larger impact is the LSI being linked to, or in partnership, with a larger (well-known) organization, such as a well-established NGO (e.g., KR, SN) or a municipality/business (e.g., HH).

As in many countries, Sweden’s public sector is limited by budget and time constraints. The economies among Swedish municipalities differ depending on the resident’s socioeconomic situations. E1, which represents a municipal city district, captures how the action space of individual civil servants can impact interest in and relation to LSIs:

*‘The problem is that there is seldom room for more within the commissions of individual civil servants. There is a lack of time and mandate to try new things. How an initiative is approached is very much dependent on the person contacted. The municipalities must be able to support sustainability initiatives, not always financially, but with space, knowledge, promotion, and without being held responsible. To make it happen there must be political support’.*

### The funding bottleneck

In the Stockholm region, the conditions for developing and sustaining LSIs are characterized by many barriers. One of the most commonly expressed challenges concerns funding, which has also been highlighted in among other fields, sustainability transition studies (e.g., Seyfang and Smith 2007; Kern 2012; Durrant et al. 2018). Many of the LSIs that participated in this study were struggling to find support for continuation and, if they did not find it, they would have to close down. Funding difficulties were found related to four main aspects: very little capacity for municipalities to support LSIs financially, lack of knowledge about regional and national funding opportunities within the LSI, lack of fit between the frames of these funding programs and the LSIs’ forms and activities and the LSIs’ lack of capacity, time and skills to apply for existing funding.

Both lack of knowledge about, and ill fit of, funding programs were found to be related to very limited connections between the local LSI level and the main regional and national funding sources. OV stresses that *‘What is needed is a better overview of actors that do things that are beneficial to the society and ask them what kind of support they need’.* Without such an overview, funding programs are not matching the LSIs’ interests and activities and are often very demanding both to apply for and to administrate, as captured by E2: *‘You can always apply for funding, but it is often complicated, and small actors need assistance; therefore, initiatives stay as just small initiatives’.* Similarly, BM stated: ‘*For the smaller organisations, it is too complicated to apply for money, and, if you get [it], 60% of the project time goes to administration’*. The funding programs are also often structured so that they require the initiative to have a certain organizational format, e.g., being a civic association or being part of a multi-actor partnership including the public sector, such as municipalities. This might be problematic, as expressed by E3: ‘*The support is given to those who already have contacts and know the system. The public sector is not so good at recognising local initiatives’.* BM concurred: ‘*Today the Swedish system for financial support results in that the money gets stuck in a funnel*—*it is only distributed to the larger organisations’.*

Besides the public funding actors’ low capacity for recognizing LSIs, the LSIs themselves partly attributed the disconnect to LSIs not being clear enough and not packaging their approaches in a communicative way so that their contributions to societal benefits are understood and can be acknowledged. During the mapping of LSI characteristics, and specifically their objectives and activities, it became clear that they contribute to the fulfilment of many ambitions and goals expressed in the Stockholm regional and local authorities’ environmental policies. E3 also realized this and said: ‘*The initiatives’ activities are working with things that relate to the National Environmental Quality Objectives, which are a regional responsibility, and therefore they potentially should support them financially’*.

The limited time frame of funding was another restriction highlighted by the LSIs. It is relatively easy to get start-up funding for up to approximately 3 years, but there are no strategies or structures for how to sustain an LSI after this initial phase, even if the LSI has proven very successful. The need for better connections between the actors with the long-term mandate and the LSI realities was captured by E2: ‘*As long as the economic logic is not there, the initiative will [be unlikely to] survive; there is a need of economic benefits in this. There is no antagonism between the large*-*scale and small*-*scale, but the small*-*scale initiatives need large*-*scale structural support to have long*-*term impact’*. Many of the LSIs that participated in this study were at the end of this initial phase and were spending most of their capacity finding ways to continue their work. During the project’s time frame, HP was closed, which seemed to be unrelated to its forms or activities, but to the need for long-term financial support and security: ‘*There are so many [who] believe in the initiative and would like to contribute and support…the growth of HP, however not in financial terms. We have realised that these kinds of initiatives need some money for administration, not much, but to keep it going’*. This situation can be described as a ‘projectification trap’, in which a lot of resources are wasted in short-term projects without a clear strategy for how to bring the knowledge further (approaches, tools, innovations, trust, social capital), and there is great risk of reinventing the wheel (Sjöblom and Godenhjelm 2009; Ehnert 2018b). OV expresses frustration over the waste of social and financial capital: ‘*Many great initiatives in this city die because key persons have to struggle to pay their rent. I think this is great waste of power for transition*’.

### Vulnerabilities

Even if several of the LSIs have grown in terms of active participants, number and diversity of activities and geographical coverage, most remain small and are seldom upscaled. One barrier to more regional-level involvement for sustainable development transformation is the resulting high degree of vulnerability, in which the total capacity of an LSI is dependent on the will, energy and engagement of a few persons, often on a voluntary basis. This severely limits the ability to apply for funding and to interact by anchoring, partnering and collaborating with other actors, locally or regionally, both of which are key mechanisms for larger-scale impact (Ehnert et al. 2018b). The tension between activities and reaching out was expressed by HP: ‘*It is difficult to make ends meet; there are so many collaborations, and it is utterly important to not lose sight of the core activities in the midst of all collaboration possibilities’*.

A similar situation was found in the public sector. New ways of thinking, organizing and doing are driven by one, or a few, persons who extend the interpretation of their assignments and supposed prioritizations to include more innovative approaches and partnerships. E1 says that ‘*Each time you talk to a representative from an initiative, you feel that you already have fulfilled your assignments and that there is no room for more. However, we can support without being [a] driving force. Today [initiatives are] very dependent on personal engagement of the civil servant*’. KR, which, over a long time, has managed to integrate a new way of dealing with large-scale green infrastructure by inter-municipal collaboration (Frantzeskaki et al. 2017) stated that ‘*From the beginning, it was very hard for the civil servants to devote time for the collaboration, and the engagement [depended] on their personal interests*’. This indicates that, even though the different actors in Stockholm express high ambitions of sustainable development, the region is still very dependent on personal engagement, willingness and courage to explore new approaches. Such dependency means a high degree of vulnerability in which a small change in someone’s life circumstances might risk the whole process of transformative capacity building, where trust-building, long-term learning and knowledge management are very sensitive.

### A disconnected region

The most pressing need highlighted by many of the participants was to create connections between actors—across sectors, across types of actors and across levels. Stockholm’s MLG seems to be disconnected, and therefore, a large part of the capacity for navigating sustainability challenges is lost. This confirms findings in other studies of the Stockholm region, e.g., water governance (Nykvist et al. 2017). During the research process, many LSIs expressed appreciation of the project’s added value because it provided them a platform on which to meet and discuss, and many of them were not aware of each other, especially not across sustainability domains. For some domains, e.g., food and green space preservation, networking has started (e.g., StS and GV), but, at the same time, the LSIs’ capacity to engage in collaborations is balanced with their capacity to have activities. StS explained this: ‘*What is missing is time for networking, since gardeners are active people who would rather cultivate than attend meetings’.* HP similarly stated: *‘It is very hard to have capacity for all collaborations…the organisation needed; we almost have to put on the brake’*.

The lack of connections between LSIs and municipalities was also expressed by many participants. One of the barriers discussed was the municipalities’ organization into domain-specific divisions, while many of the LSIs work across multiple domains—e.g., EA working with gardening, food production and education, while addressing social segregation. Often, there is no clear connection point where the LSIs can start communicating with the municipalities. The participants expressed a need for ‘sustainability translators’ who can facilitate communication with municipalities about, for example, regulations, formalities and opportunities. The disconnect was found a bit surprising, given the long-term Swedish tradition of involving and interacting with civic associations, which can be interpreted as good grounds for trust, communication and collaboration. However, many LSIs take other forms than the traditional association—some because they think the traditional format is not useful, and others because they do not have the capacity it takes to organize such an association. Even if a municipality interacts well with local civic associations, it might not recognize, or know how to approach, these other LSI forms.

Another disconnect in Stockholm’s MLG was between the municipalities themselves. Stockholm’s regional authorities have, over the past few years, increasingly engaged in creating platforms for supporting dialogue across municipal borders. The aim has been to identify potential for synergies in action and to handle the region’s common urban challenges. However, there are still very few formal incentives for engaging in inter-municipal collaborations, which is evident by the long-term work of the LSI KR, where a partnership of NGOs and a few regional and municipal civil servants slowly managed to convince the municipalities to engage in lengthier and more formalized collaboration (Frantzeskaki et al. 2017).

### Constituents of a new mode of governance

The Stockholm region hosts great potential for supporting city wide transformation toward sustainable development, due to;

- the large diversity of different LSI in terms of format and in terms sustainability domains, geographical range and activities of as shown in this study
- the high ambitions of sustainable development expressed in both regional and local policies (Borgström and Svensdotter 2015).
- the long-term tradition of working with engaged citizens organized in civic associations (von Essen et al. 2015).
- the efforts of networking and collaborations between actors and across levels and sectors, becoming showcases for other settings (Borgström and Svensdotter 2015).
- the expressed need among several of the municipalities to find new ways of navigating wicked sustainability challenges (Borgström and Oreskovic 2016).

However, this potential is severely hampered by the disconnected governance structure, where many actors are working in isolation, in both a horizontal and vertical sense, and often based on short-term project funding.

From the trans-disciplinary process, several proposals improving the situation and making better use of the LSIs in the Stockholm region were discussed and later formulated into a roadmap (Table 2, Borgström and Oreskovic 2016). The jointly identified core challenge in the region is to decrease the fragmentation and isolation of levels, sectors and actors, which translates to the need for inclusive governance and working across connecting diverse actors and levels as proposed in literature (McCormick et al. 2013; Frantzeskaki et al. 2016; Wolfram 2016a; Buijs et al. 2016; Gorissen et al. 2017). Generally, the suggested strategies involved developing clear and transparent channels for enhanced information flows; establishing fora to facilitate exchange, mutual learning and increased understanding between actors; and flexible and hence adaptive structures for long-term financial support to allow different LSIs, and other actors, to participate in these collaborative ventures (Table 2). The main suggestions directly relating to balancing diversity and connectivity are presented below.

**Table 2:** Proposals for how to better integrate the LSIs into the MLG of Stockholm region (based on Borgström and Oreskovic 2016)

Systems-thinking	Increasing the awareness of how the parts of a system are linked to each other and to other systems, e.g., the interaction between the ecological, economic and social dimensions; understanding and responsibility for the flows of materials and energy
LSI coordinator	Establish a municipal civil servant with the responsibility to oversee and evaluate the LSIs and how they relate to the municipal sustainability goals. They should also act as networker by connecting different actors to each other
LSI pilot	Establish a municipal civil servant who supports LSIs to find the right contacts within the municipal administration and assists in practical matters.
Community Development Group	Establish a municipal working group with the tasks to integrate, make visible and support LSIs and other local sustainability efforts in municipal activities. This group includes initiative pilots and initiative coordinators
Sustainability council	Establish a regional body with representatives from municipalities, LSI, other businesses, culture sectors, politics etc., which oversees the region’s sustainability efforts, acts as a consultative platform and a forum for long-term strategic work
Integrated vision	An important task for the Sustainability Council is to develop an integrated vision of how sustainability in the region can be implemented to benefit from the various actors’ expertise and opportunities, including LSIs
Facilitators/process leaders	In order to establish and sustain cross-actor/sector/level dialogues and collaborations there is a need for acknowledging and recruiting specific competences in facilitation and process design and leadership. These are not project managers, coordinators or representatives but rather individuals working to support them in collaborative processes and dialogues
Sustainability centre	Establish physical locations where sustainability actors and alternative practitioners can converge in a concrete way; where local actors are given the opportunity to get in touch with LSIs
Open space	Allow for physical spaces for supporting creativity where sustainability actors can experiment and try out new ideas and alternative methods. These sites can be temporary which removes certain regulatory frameworks to allow for creativity
Online venues	Establish and maintain online platforms, networks and forums where LSIs can be seen and interacted with, and where knowledge, resources and inspiration can be shared
Database of funding possibilities	Provide a regional list of funding opportunities for LSIs that is continuously updated. The Sustainability Council could be a potential host of the database
Collection of learning examples	Create a catalogue of sustainability approaches to learn from both successful and less successful but learning examples
Cultural sector	Acknowledge that the cultural sector has the expertise and tools to raise complex issues, supporting challenging dialogues and highlighting issues in new ways. Representatives from the cultural sector are an important part of the Sustainability Council, Sustainability Centre, and as process leaders
Planetary stewards	As a way to acknowledge the importance of LSIs and other sustainability actors in the region the title ‘planetary steward’ should be established and used
Business models for sustainability initiatives	Since LSIs commonly work with collective benefits they need new models; structures and processes; for how to work in the long-term perspective

There is a clear need to improve the municipal structures and processes for working with different LSIs—different in their thinking, organizing and doing (e.g., those working across sectors or having innovative organizational forms). The first step is to increase awareness of LSIs’ existence within the municipalities and how their concrete, local activities contribute to the implementation of policy goals and strategic programs. The next step is to reshape the structures of communication, involvement, funding, etc., to allow for different LSI forms and, at the same time, to acknowledge challenges regarding responsibility, representation and accountability. Wolfram (2016b) highlights the importance of working across levels by recognizing, acknowledging and sustaining intermediaries. In the Stockholm case, as well as in other cases within the ARTS project (European Commission 2017), the importance of ‘translator’/’networkers’ who have a long-term experience of many different contexts and organizations, and who can carry knowledge and understanding between actors in an MLG, was emphasized. This role and its versatility in the MLG context have been acknowledged also in the resilience literature as ‘scale-crossing brokers’ of importance for connections between actors (Ernstson et al. 2010), in the transformation literature as ‘change agents’ that are instrumental in all phases of transformation (Westley et al. 2013) and in socio-technological transitions as ‘boundary-spanners’ that facilitate exchange between actors (Smink et al. 2015). However, this is presently an informal capacity, and one proposed strategy was to formalize such a role by establishing municipal LSI coordinators. These roles would include having an overview of the LSIs in the municipality, developing support that fits these LSIs and guide the LSIs in their contacts with the municipality, businesses and with other actors.

Another proposed strategy was to establish long-term, local sustainability centers—spaces where LSIs can meet each other, residents can meet LSIs and LSI activities can take place and, therefore, become more visible. In addition, this would also be a well-known place where other sustainability actors, e.g., local and regional authorities, would know they could find and interact with LSIs. These centers could function as locally based extensions of urban living laboratories (Bulkeley et al. 2016). This is clearly a strategy for increasing connectivity, but, instead of each LSI and civil servant establishing and sustaining their own networks and networking, which is a resource demanding activity, the connectivity would be developed by these external centers. Such centers can also constitute interfaces where needs for diversity by a certain degree of LSI autonomy are balanced with needs for connectivity to enable upscaling, replication and support as part of increasing the use of LSI knowledge in the larger city region.

There was also an expressed need for easier access to physical places where new ideas can be tried and tested. The participants called these ‘free spaces.’ For this, changes in the allowances of certain open spaces and temporarily unused public buildings were considered necessary. E1 described it as follows: ‘*The municipalities must be able to support sustainability initiatives, not always financially, but with space, knowledge, promotion, and without being held responsible. They must become less nervous regarding how public spaces are used. To make it happen, there must be political support’*. Such reconfiguration of urban space has also been highlighted as of importance for the local emergence and spread of innovations (Radywyl and Biggs 2013; Wolfram 2016a). The proposed establishment of local centers and free spaces is concrete actions that potentially enables experimentation, learning and embedding into larger governance structures and processes, as well as cross-scale/actor/sector interactions, which have all been suggested as components of urban transformative capacity (Wolfram 2016b).

For securing a more long-term strategy to support LSIs and better connect their work to the regional-level ambitions, a sustainability council was proposed, including representatives from regional actors, municipalities, LSIs, businesses, the cultural sector, etc. One important task for this council would be jointly developing, and continuously updating, an integrated vision for sustainable development aimed at aligning different actors’ activities and, thereby, clarifying how each actor contributes or could contribute and furthermore, identify gaps and emergent new issues that needs attention.

## Concluding remarks

This study’s findings highlight the embedded tension in the components of urban transformative capacity—the tension between the need for cross-scale interactions, coordination and collaborations, and the need for diversity and autonomous communities of practice for place-based experimentation and creativity. The following statement from one of the participants well illustrates the tension: *‘We need to collaborate, work together and find synergies to move from initiatives and project into more long*-*term activities without losing quality or creativity’.* Therefore, an important part of transformative capacity is to create enabling environment for new governance modes that include new approaches to collaborations (Gorissen et al. 2017) that moves away from the idea that any kind of collaboration is good and leads to progress (Bodin 2017). As shown in this study, this is especially important in the urban MLG setting.

Given the diversity of LSI forms (Ehnert et al. 2018b) different initiatives have different capacities to engage in collaboration, which must be carefully recognized to avoid unconscious exclusion of innovative approaches developed by new constellations of people. To address what kinds of collaborative efforts are needed within an urban MLG, tools must be developed that can assess the collaborative capacities among various actors and, from that, develop purposeful and adaptive collaborative formats. An important part of the collaboration dynamic is the intermediaries whose important role in supporting transfer of knowledge and understanding between the actors has been pointed out by several scholars and in different fields (Hahn et al. 2006; Ernstson et al. 2010; Wolfram 2016b). However, the next step, and part of new governance mode, is to proactively support the establishment of such intermediaries, which was included in several of the suggested measures from this study.

On the other hand, the potential of LSIs lies in their freedom to develop and try innovative, place-based solutions—to let local challenges and local knowledge evoke creativity. From a municipal and regional perspective, the LSIs in Stockholm provide a large diversity of approaches, good examples and test beds to sometimes rather similar challenges played out in various local contexts. In a way, this is also what the Swedish decentralized MLG seeks to achieve by strong municipal power. To safeguard LSI diversity, it is necessary to shape funding programs that are flexible enough to include a large variety of LSI formats and capacities. Structures and processes of funding were identified as critical to the LSI capacity, but have been surprisingly little addressed in the MLG and transformation literature. Hence, more attention needs to be paid both in research, policy and practice what the old and new modes of governance imply in terms of resources. Another aspect of diversity is to offer support to front-runners, like the LSIs, while including the public—ensuring large scale transformation. This is an important challenge and responsibility for local authorities, like municipalities.

A dominating focus on coordination and collaboration to reach common understandings and decision-making risks disregarding specific, in-depth system knowledge (both scientific and experience based) that is essential for prioritizing purposeful and contextualized activities for sustainable development. A capacity to continuously zoom in and zoom out between the general and the specific is, therefore, necessary for progressive collaborations. However, the opposite was expressed by several well-experienced networkers partaking in the project who stated that most collaborative efforts did not fulfil their ambitions. Therefore, to achieve purposeful and progressive collaborations, it is necessary not only to focus on the desired outcomes, but also to recognize the importance of process goals (Westin et al. 2016). If the collaborative components of transformative capacity are to be realized to release the capacity of MLG settings, knowledge and skills in process design and facilitation must be further developed and better integrated into the interventions.

The Stockholm region has the potential to transform toward sustainable development, but it has not unlocked its capacity. The multi-levelness is there; the diversity is there—but there is still a need to further evaluate its governance structures and processes and seek and try new models that carefully balance diversity and connectivity. As one of the respondents phrased it: *‘We have very little power, but freedom to act; they [decision*-*makers] have lots of power, but limited freedom to act’.*

## Electronic supplementary material

Below is the link to the electronic supplementary material.

Supplementary material 1 (PDF 690 kb)
