# Supplementary material for: Balancing diversity and connectivity in multi-level governance settings for urban transformative capacity
Source: Ambio. 2019 Feb 15;48(5):463–77. doi: 10.1007/s13280-018-01142-1 (PMC6462285; doi:10.1007/s13280-018-01142-1)
Supplement: Supplementary file 1 — Supplementary material 1 (PDF 690 kb) [file 13280_2018_1142_MOESM1_ESM.pdf]

Ambio

Electronic Supplementary Material
This supplementary material has not been peer reviewed.

Title: **Balancing diversity and connectivity in multi-level governance settings for urban transformative capacity**

Author: Sara Borgström

APPENDIX S1 Participants in the trans-disciplinary research process

Table S1 List of key informants contributing to the scoping process

ID	Description
KI1	Representative for the transition movement in Stockholm/Sweden, LSI OV (table 1)
KI2	Representative from the largest adult education association in Sweden, Studiefrämjandet
KI3	Representative from the Stockholm County Administrative Board, Environmental planning division
KI4	Representative from a sustainable development consultant firm
KI5	Representative from Stockholm County Council planning division
KI6	Representative from Stockholm County Council planning division, green structure issues
KI7	Representative for the network of urban gardeners in Stockholm, LSI StS (table 1)

Table S2 Experts included in the interview study

ID	Description
E1	Civil servant working with sustainability issues at the city district level within Stockholm municipality.
E2	Civil servant working with sustainability issues at at the Stockholm County Association of Local Authorities (KSL) that represents all 26 municipalities in the region
E3	The coordinator of the regional work about the National Environmental Quality Objectives at Stockholm County Administrative Board
E4	Presently independent consultant but with long term engagement within the Swedish Association for Nature Conservation in Stockholm (the largest environmental NGO in Sweden).
E5	Presently independent consultant but with long term experiences from different political organisations in Stockholm region (Värmdö and Tyresö municipalities, Stockholm County Council, Mälardalsrådet)

Table S3 Participants in workshops (WS1-2) and focus group meetings (FG1-3)

Meeting	Participants
WS1	LSIs: StS (KI7 table S1), ES, EA, HH, HP, OV (KI1 table S1), RF, LT, SN, MV, KR, KS, GV (table 1)<br>Nacka municipality, Albaeco (sustainability communication association)
WS2	LSIs: MV, EA, LV, RF, SN, StS, KS (table 1), Höj rösten*, Bagarmossens resilienscentrum*, Provins mat* (* = not part in the interview study)<br>Regional authorities: Stockholm County Administrative Board (E3, table S2), Stockholm County Council Planning division, Mälardalsrådet<br>Municipalities: Nacka municipality, Sollentuna municipality

	Key informants/Experts: KI4, E3, E5 (table S1, S2)<br>Private sector: White arkitekter, Guringo, Origo/Resource lab, Stormie Poodle, Nyréns arkitektkontor, Urbio<br>Cultural sector: Färgfabriken, 5 independent artists
FG1	LSIs: StS (KI7 table S1), LT, OV (table 1), BeeUrban* and Provinsmat*<br>(* = not part in the interview study)
FG2:	Municipalities: : Huddinge municipality, Haninge municipality, Södertälje municipality (represented by ES LSI, table 1) and Stockholm municipality (represented by HH LSI, table 1)
FG3	Regional authorities: Stockholm County Administrative Board (E3, table S2), Stockholm County Council Planning division

Description of LSIs participating in the interview-study

(Adapted from Borgström and Svensdotter 2015, see also Figure 1 and Table 1)

AM - Adelsö Mobilsamåkning / Adelsö Mobile Carpooling

AM is a **single LSI** that is part of a larger network of several initiatives that share the common aim of supporting local businesses and increasing tourism in an environmentally sustainable way that was initiated 2011. The ten people that started the network listed associations, companies, people, and all the things you could do, e.g. a visitor's centre. The municipality did however not really understand and some of the members thought it to become too big. However, AM was realised by the establishment of a carsharing system that enables people who have a car and people who need a ride to connect and save CO_2, time, and money on co-commuting. This alternative mobility also aimed to limit the pressure on the already limited transportation infrastructure (both road quality and public transportation structure). The carsharing system using a free mobile application was replicated from another community in Southern Sweden and in 2013 AM had approximately 100 users and was registered as a small business. Even if acknowledged by the municipality as an excellent example of sustainable development, their applications for long-term support and funding beyond pilot project failed and at the time of the interview the local engagement was fading.

StS - Stadsodling Stockholm / Urban Agriculture Network Stockholm

StS is a **networking LSI** that started in 2013 and aims to connect urban gardening initiatives in Stockholm. The founders found out that many urban gardens were temporary and disorganised but sharing the difficulties in communicating with the municipality about their interest in gardening on public space. The first step was to create a map of all initiatives and thereby facilitate match-making. They wanted to be a voice, influence and be a contrast to the city as well as a platform of sharing of knowledge and experiences and other kinds of support. The network, which is now organised as a non-profit association and is communicating by a Facebook group and an active partner in several urban gardening arrangement in Stockholm. The activities are based on voluntary engagement.

ES - Ekomat Södertälje kommun/Organic food in Södertälje municipality.

ES is a **municipal LSI** that was formally launched in 2010 when the municipality decided on a meal policy to increase the proportion of organic food of the public meals (e.g. to child and elderly care facilities), which includes 89 kitchens that provides 24000 meals daily. There was already a high degree of awareness among the municipal residents, both about the environmental impact of food production as well as more sustainable alternatives. It was from them that the demand for change came, which was very early compared to most of Sweden. A nutritional manager was recruited to the municipality with the responsibility to run the kitchens and thereby controlling all purchases and with the ability to use the meals to educate the municipal employees towards the new policy. The municipality has since received much national and international attention for its work with climate friendly and organic food public meals and this has become an important part of the city identity and branding. The LSI has been scaled by being part of the EU project BERAS implementation that further refined the concept of Diet for a Green Planet and kitchen certifications. More lately also into several projects for increased job opportunities in the green businesses focusing on local food production and in the five-year EU project MatLust based in Södertälje that supports small and medium sized businesses in the sustainable food sector of the Stockholm region.

BM - Bondens marknad/Farmers market.

BM is a **regional LSI** and was the first farmers market in Sweden and Stockholm started in 2000 (with linkages to the US initiatives starting in 1970s and spreading across Europe). The aim was to provide an alternative market for providing direct contacts between the urban consumers and local food producers. The background was that with the rapid pace and large-scale food production industries local specialities and small producers disappear, even though there is a demand for them. The start of the initiative was funded by the Farmers National Organisation in Sweden. The national farmers market is run by the entrepreneurs themselves and each of the local markets is run by a local economical association with local producers. Besides being a market, it is also a network of local producers. The rules are that the producers must have grown or bred what is sold in the market and that the farms cannot be located more than 250 km away from the market place. Today there are 15 farmers markets around Sweden and two of them are located in Stockholm.

EA - Ekoodling Akalla/Hjulsta /Organic gardening Akalla/Hjulsta.

EA is a single LSI with the vision to give people in the Stockholm north western suburbs a chance to show their skills and knowledge, give access to organic and healthy food and at the same time create a common meeting ground. It is combining ecological sustainability by locally producing food with justice in terms of empowering the residents to have a voice, access to land and thereby being able to act glocally. A civic association was created through the local branch of the one of the Swedish study associations for adult education and the local 4H farm in 2013. The association established an urban garden with support from EU-funding and the Employment Agency supporting with workforce. Of the produce 20% was for public use and 80% for markets

and restaurants. Later they were evicted by the municipality from the premises and changed location. The initiative has been followed by conflicts and a growing mistrust to the local authorities. Today they have 15 members and lease 2000 square meters from the Stockholm municipality and on a voluntary basis organise gardening, study groups, markets, dinners, guerrilla gardening and interact with locals.

HH, HP - Hållbara Hökarängen och HOPP!/Sustainable Hökarängen and HOPP!

HH was a **municipally lead LSI** coordinated by the municipal property owner Stockholmshem 2011-2015 in the city district Hökarängen owning 3000 apartments and 500 premises. The project was based on a mixture of in-kind funding from partners in the project as well as external funding. The main aim was to identify methods and solutions that works and could be spread within the company and within the municipality at large. They realized that they needed to have a more holistic approach "than establishing another soccer game area" and therefore launched the initiative focusing of sustainability in a broad sense for community building. A resident dialogue and the goals of the company defined three core areas for action; energy efficiency, core area development and artistic businesses. In the next phase it also included behavioural change and climate impact and in collaboration with innovation actors they started exploring how to engage residents in sustainability. They had a very supportive approach, being aware of the need for establishment of local initiatives that could take over when the project was finalised. One of those was HP which sprung from HH providing meeting place and matchmaking between engaged residents in the city district. HP was a **single LSI** based on three persons engagement and that provided courses in community sustainability practices, e.g. permaculture gardening and transition dialogues. HH have high hopes that HP will be one of the lead initiatives to continue develop the Hökarängen city district and they supported them with start-up funding. They had a lot of ideas about continued community building but despite large efforts they could not secure funding for realisation of these ideas and was therefore closed in 2016. The insights from HH has been used in similar initiatives by Stockholmshem in other city districts in Stockholm.

Hållbara Hökarängen
- ett utvecklingsprojekt inom Stockholmshem

HOPP!
HÖKARÄNGENS OMSTÄLLNING & PERMAKULTURPROJEKT

OV - Omställning Värmdö/Transition Värmdö

Transition Värmdö is a **single LSI** which is also part of the international Transitions Movement and share the same idea – a public movement locally addressing the need for systems thinking on transition and working for a sustainable and resilient society, providing local solutions for the many challenges ahead, including economy, energy, and ecology. The founder of Transition Värmdö was engaged in the Swedish Society for Nature Conservation and took a course about transition leading to establishing a local transition group. They work locally with creating sustainable societies based on: Conservation of biological diversity, Networking for common projects, Gardening for increased local food security, Green energy production, A local economy where the money is circulated in the surroundings and invested in local production. The arrangement of the event "Framtidsveckan" (A week for the future) annually for three years aimed at creating a locally based dialogue on solutions to handle future environmental challenges by inviting 50-60 actors with different activities and was visited by about 2000 people. However, this was a very demanding activity that did not lead to the results the initiative

had hoped for, therefore they scaled down and are now focusing on local community sustainability.

RF - Refo /Refo – Remake and Reuse of Clothes.

Refo is a **single LSI** - a company that works with redesign and remaking of textiles, clothes and other materials. Refo grew out of another business that the founder had been running for 20 years and was founded on the basis that there are families who does not afford to buy clothes for their children, at the same time there are many people that too much clothes, it is not only environmentally wasteful not to use them, but there are people who need them. In addition, there is also a growing proportion of people under 30 years old that stands outside the labour market with a need for linguistic training, knowledge and work, etc. Refo is selling its products in a showroom, through 15 small shops and through a website. People can donate material, and the items will either be remade or packaged and distributed to children and teenagers through e.g. local nurses and midwifes in suburbs both north and south of the city. Primarily young people who are out of employment do the remaking and redesigning. This creates local hubs of people who are designing and reselling. Altogether more than 100 people are involved in the business.

LT - Lekträdgården – Lek Odla Väx / The Play Garden – Play Grow Cultivate

LT is a **single LSI** that in 2012 was established within a larger organisation the foundation Rosendal's Gardens, and initially based on funding from the Swedish Inheritance Fund for a children's project aiming at increasing the understanding of where food comes from, the context, and nature's processes and seasonal changes. A project manager and two gardeners with a pedagogical background was recruited and since the start more than 1000 children and pedagogues from the Stockholm municipality have been engaged, besides children participating in activities during the general garden events. The interest for the initiative and their pedagogy has been large and they have published two educational books presenting their methodology. From supporting school classes on site, the activities today also include courses, single lectures and inspirational meetings for pedagogues as well as consultancy for supporting school yard gardening. The initiative is non-profit where the incomes from different activities is used for running and developing the initiative.

SN - Storstockholms naturguider/Greater Stockholm Nature Guides

SN is a **regional LSI** that since 2003 has organized guided nature tours for the public almost every weekend the year round and tours for specific target groups, e.g. daytime tours on weekdays for parents on parental leave or unemployed. The motivation for the initiative is that if you do not know about nature it is hard to like it and care about it. To raise

awareness of the many accessible green areas in Stockholm as a leverage to more general environmental consciousness is the main goal. The initiative is run by the regional branch of the Swedish Society for Nature Conservation but with financial support from municipalities, regional authorities and also from organising conference and business events. The guides are authorized as any other guide in Stockholm and get payed for preparing and performing the tours. The participants must pay a fee to join the tours and the incomes complement financial support from other actors.

MV - Miljöverkstan Flaten/ The Environmental Workshop Flaten

MV is a **single LSI** that started in 2011 around a swimming site in a nature reserve south of Stockholm – Flaten. The place was falling apart but the founders saw that the place could offer much more to the residents and they wanted to build bridges to the site by giving the children and youngsters a feeling for nature. A group of local engaged actors started to discuss revitalisation of the area with the City District Administration that was very positive and provided funding when other applications had been turned down. The Environmental Workshop was formed and attracted interest. Mostly based on external funding they work with sustainability by place-making, focusing on local kindergartens, school kids and teenagers in various projects using the area and local resources. The LSI is today an economic civic association with support from among others the Swedish Inheritance Fund but has in recent years been forced to downscale due to lack of financial resources, despite expressed interests from the municipal politicians.

KS/KR - Mellankommunal samverkan Stockholms gröna kilar/Municipal collaboration of Stockholm green wedges

From a perceived need to coordinate the municipalities around the green wedges of Stockholm, NGOs representing environmental protection, tourism, outdoor recreation and cultural heritage formed the network "Protect the green wedges of Metropolitan Stockholm" in 1996, KS **regional LSI**. KS was triggered by a report about the values of the ten green wedges launched by the Stockholm County Council and this report was used by the KS in local and regional urban development debates. The ideas of KS were then concretised in 2006 when the KR **inter-municipal LSI** was established by the KS partners and the Stockholm County Council engaging 6 municipalities and NGOs in one of the Stockholm regional green wedges – the Rösjö green wedge. The first steps included joint study visits, workshops, creation of a visitor's map, and building networks with regional authorities and other external key actors and eventually a platform for collaboration was created that also gained political support in the municipalities. At a certain point of concretising, including work plan and budget, the politicians realized that the municipalities would be the leaders of this initiative. The KR was then reorganized, and the formalization process started. It has gained attention and the insights from KR has been spread to other green wedges in the Stockholm region, but so far, no formal collaboration similar to the KR case has been established.

GV - Ny Grön Våg (Ny Grön Stad) / New Green Wave (New Green City)

GV is a **networking LSI** (now named New Green City) was founded in 2011 as a network of initiatives in Stockholm's regional green wedges, from an initiative of key actors within the regional branch of the Swedish Society for Nature Conservation and WWF. From the reality of rapid urban development by densification in Stockholm, they saw the possibility to have a larger impact by networking and exchanging knowledge among the growing number of local initiatives protesting to safe urban green space from exploitation.

The network aims to support these groups by better distribution of the knowledge and experiences among the 18 current partners being both local protest groups and formal associations at local and regional level. The network is based on in-kind, voluntary engagement from member partner organisations.

APPENDIX S2 - Interview guides

Interview questions for representatives of local sustainability initiatives
1. What problem or what possibility initiated this initiative?
2. How did the initiative start?
3. Were there other, previous, initiatives that had a positive (e.g. inspired) and/or negative (e.g. competition) impact on your initiative?
4. Has the initial idea of the initiative changed in character or developed in some way)? And if so, in what ways and why?
5. In your view, what has been greatest a) success and b) setback of this initiative so far?
6. Has the initiative grown in number of participants or budget?
a) What factors have contributed to growth or decline?
b) If the initiative has grown, what have been the effects of that growth?
7. What is the core team of active people and how has it changed of time?
a) What are the commonalities between people in the team? b) What keeps the team together?
8. What other people can tell about this initiative – internal or external?
9. Have you applied for and received economic or other substantial support for the initiative?
a) From private, public, local or regional actors? b) What other resources are important to you and in what ways?
10) In what ways do you collaborate with other actors within the city region or beyond the city region?
a) What motivates this collaboration?
b) How have the forms of collaboration changed over time?
c) Has collaboration been important to your initiative?
d) Do you have experiences of conflicts with other initiatives?
11) Has your initiative been part of creating or adjoining in alliances or networks, formal or informal?
a) What was the main purpose with this networking?
b) In what ways have the network impacted the city?
12) How do you work for engaging active or members in the initiative?
13) Do you organise activities aiming at engaging local residents or other actors in the local area, that are not active in your initiative and what are the reasons for this?
14) What kind of people are engaging in and/or supports your initiative?
a) How inclusive do you think your initiative is?
b) Is it important for the initiative to be inclusive?
15) Do you have the ambition to reach out to the general public with your initiative and if so, why?
a) Who is your main target group?
b) What channels do you use for reaching out?
16) Which actors in the Stockholm region do you try to influence?
a) Why these actors?
b) What kind of methods do you use?
17) What are the relations between the decision-makers and your initiative?
18) Please exemplify factors that you think have been a) most helpful/positive and b) most discouraging/negative and c) how these have changed over time.
19) What strategies at city regional level do you think would accelerate the transformation towards a more sustainable development in Stockholm?

Interview questions for experts

1) Which ones of the following initiatives do you know about?
2) In what ways do national/regional/local and other institutions support local sustainability initiatives in Stockholm and how well is this working in your view?
3) If any, what kind of improvements do you see important in the present support system?
4) According to you, what is the degree of collaboration between initiatives in the region?
a) If there is limited collaboration, do you think there is a need for more?
b) If more collaboration is needed, what is needed to accomplish this?
5) How much do you think local initiatives in Stockholm have contributed to the sustainable development?
6) How social inclusive do you think the initiatives are? How can the inclusivity be increased if considered needed?
7) According to you, is environmental sustainability a prioritised issue at the agenda of the decision-makers in Stockholm?
8) How effective do you think the local policies and implementation have been in recognizing sustainable development?
a) How inclusive has the policies and implementation been?
b) Are there segments of the society that are less involved or even excluded?
9) In the Stockholm region – how well is coordination and collaboration between the public, private and civic sectors been in order to support local initiatives for sustainability? How can the collaboration be improved?
10) What possibilities do you see to accelerate the transformation toward sustainable development in the Stockholm region and what barriers do you see?

APPENDIX S3 Coding scheme
*** Part of the analytical framework, but not included in the findings of this paper.**

VARIABLE	EXPLANATION
**Geographical scale**	**Indicates what geographical scale the condensed MU refer to**
Local	Neighborhood, local or city district level.
Regional	Any factors/developments within the region which influence ways of organizing (structures), ways of thinking (cultures) and ways of doing (practices).
External to region	Any factors/developments outside the region which influence ways of organizing (structures), ways of thinking (cultures) and ways of doing (practices) in the city-region.

**LSI activity**	**Information about any activity (new ways of organising, thinking, doing) of the selected LSI.**
Motives	Motives and/or problem that initiated the LSI.
Ambition	Ambition and/or goals with the LSI
Genesis and development	Why and how the LSI started and has developed since.
Activities	What activities the LSI has.
Outreach	Outreach and communication activities
Inspiration	Inspiration from others
Sustaining	How the LSI is sustaining its organisation and work.
What is needed	What is needed for LSI sustenance and development

**Factors impacting LSI progress and impact**	**Factors and/or strategies used by or influencing/hindering LSI to activate/contribute to urban sustainable development.**
All below codes were tagged as navigation strategies (positive, negative or necessities), success factors (enable the LSIs sustenance and/or development) or hindering factors (hinder or limit the LSIs sustenance and/or development)	
Resources	Financial strategies/needs and non-financial resources
Priorities	Priorities, usually of time or resources.
Structure	Internal structure and process (type of organisation, internal processes, core values)
Flexibility	Flexibility within organisation or within given mandate.
Key stakeholder	Important/vital person for the TI.
Network/ collaboration	Collaboration/networking within local areas and across the region
Competition	External competition.
Communication	Communication/dialogue/visibility
Cross-domain	Crossing different domains or arenas.

Decoy	Strategies to use one mean to reach for another, usually used as a political game.
Joy	Joy or positivity as an enabling factor.
Drainage	Drainage of overall capacity.
Disembedding	Disembedding/localising. Factors that hinders or limits the embedding.
Conflicts	Conflicts that arise either between LSIs or actors.
Trust	Trust within LSIs and to other actors.
Controversies	Controversial (e.g. in relation to mandate, overall system understanding)
Political support/Relation to authorities	Political support and accessibility, local, regional or external and overall relation to authorities/decision makers
External governance	Policies, trends. Any factors/developments outside the region which influence ways of organizing (structures), ways of thinking (cultures) and ways of doing (practices) in the city-region.

**Acceleration mechanisms***	**Overarching accelerating mechanism based on ARTS project conceptual framework (Frantzeskaki et al. 2015, Gorissen et al. 2017)**
Upscaling	Of a single initiative through growth of members, supporters or users within the region in order to spread new ways of doing, thinking and/or organizing.
Replicating	Copying the new ways of doing, thinking and/or organizing of one transition initiative by another initiative within the region
Partnering	Linking between transition initiatives within the region within the same domain or across domains in order to exploit synergies between new ways of doing, thinking and/or organizing.
Embedding	Embedding new ways of doing, thinking and organising into regional governance patterns
Instru-mentalising	Instrumentalising developments at subnational, national, transnational or European levels (i.e. in the multi-level governance context of the city-region) in order to strengthen new ways of doing, thinking and/or organising locally.
Acceleration	About acceleration and ways to accelerate
Inclusivity	Approach to diversity in the region.
